# Multi-regulated GDP-l-galactose phosphorylase calls the tune in ascorbate biosynthesis

**DOI:** 10.1093/jxb/erae032

**Published:** 2024-02-13

**Authors:** Pierre Baldet, Kentaro Mori, Guillaume Decros, Bertrand Beauvoit, Sophie Colombié, Sylvain Prigent, Pierre Pétriacq, Yves Gibon

**Affiliations:** Université de Bordeaux, INRAE, UMR1332 BFP, 33882 Villenave d’Ornon, France; Université de Bordeaux, INRAE, UMR1332 BFP, 33882 Villenave d’Ornon, France; Max Planck-Institute of Plant Molecular Biology, Potsdam-Golm, Germany; Université de Bordeaux, INRAE, UMR1332 BFP, 33882 Villenave d’Ornon, France; Université de Bordeaux, INRAE, UMR1332 BFP, 33882 Villenave d’Ornon, France; Université de Bordeaux, INRAE, UMR1332 BFP, 33882 Villenave d’Ornon, France; Bordeaux Metabolome, MetaboHUB, PHENOME-EMPHASIS, 33140 Villenave d’Ornon, France; Université de Bordeaux, INRAE, UMR1332 BFP, 33882 Villenave d’Ornon, France; Bordeaux Metabolome, MetaboHUB, PHENOME-EMPHASIS, 33140 Villenave d’Ornon, France; Université de Bordeaux, INRAE, UMR1332 BFP, 33882 Villenave d’Ornon, France; Bordeaux Metabolome, MetaboHUB, PHENOME-EMPHASIS, 33140 Villenave d’Ornon, France; University of Exeter, UK

**Keywords:** Abiotic stress, ascorbate, light, multi-regulated GDP-l-galactose phosphorylase, PAS/LOV, photoreceptor, transcription factors, uORF, vitamin C

## Abstract

Ascorbate is involved in numerous vital processes, in particular in response to abiotic but also biotic stresses whose frequency and amplitude increase with climate change. Ascorbate levels vary greatly depending on species, tissues, or stages of development, but also in response to stress. Since its discovery, the ascorbate biosynthetic pathway has been intensely studied and it appears that GDP-l-galactose phosphorylase (GGP) is the enzyme with the greatest role in the control of ascorbate biosynthesis. Like other enzymes of this pathway, its expression is induced by various environmental and also developmental factors. Although mRNAs encoding it are among the most abundant in the transcriptome, the protein is only present in very small quantities. In fact, GGP translation is repressed by a negative feedback mechanism involving a small open reading frame located upstream of the coding sequence (uORF). Moreover, its activity is inhibited by a PAS/LOV type photoreceptor, the action of which is counteracted by blue light. Consequently, this multi-level regulation of GGP would allow fine control of ascorbate synthesis. Indeed, experiments varying the expression of GGP have shown that it plays a central role in response to stress. This new understanding will be useful for developing varieties adapted to future environmental conditions.

## Teaser

In the plant kingdom, the biosynthesis of ascorbate (vitamin C) by the Smirnoff–Wheeler pathway is mostly controlled by GDP-l -galactose phosphorylase at the transcriptional, post-transcriptional, and catalytic levels.

## Introduction

Ascorbate is an essential metabolite in living organisms. As the main antioxidant, it maintains the redox state of the cell by eliminating reactive oxygen species (ROS) that are usually produced in response to biotic and abiotic stresses (Decros *et al.*, 2019). Ascorbate also plays a role in controlling the levels of ROS that are continuously produced under optimal conditions by cell metabolism, notably in the presence of light during reactions that participate in the mechanism of photosynthesis and photorespiration within the chloroplasts and peroxisomes, respectively (Choudhury *et al.*, 2018). In addition, ascorbate plays a pleiotropic role in hormone biosynthesis, the xanthophyll cycle, flavonoid biosynthesis, iron uptake, and gene expression (Pastori *et al.*, 2003; Page *et al.*, 2012; Grillet *et al.*, 2014; Smirnoff, 2018). Thanks to its high antioxidant potential, ascorbate is one of the most important traits for the nutritional quality of fruits and vegetables. Indeed, evolution in humans and a few animal species led to the loss of the l-gulono-1,4-lactone oxidase activity that catalyses the last step of the animal biosynthetic pathway (Burns *et al.*, 1957). Consequently, humans are unable to synthesize ascorbate, deﬁned as vitamin C, and must have a daily intake through the consumption of fruit and vegetables. Interestingly, there is still a controversy about how much vitamin C is beneficial for human health, but the recommended daily allowance is between 100 mg and 400 mg, and depends in general on a person’s age and physiological state.

In plants, there is a great variability in ascorbate content according to the genus and the species. A longstanding misconception is that citrus fruits have the richest contents of vitamin C. In fact, their ascorbate content (50 mg 100 g^–1^ FW) is very modest compared with two fruits from South America, namely camu-camu (*Myrciaria dubia*) and acerola (*Malpighia emarginata*), that accumulate up to 3 g and 2 g 100 g^–1^ FW, respectively (Maciel *et al.*, 1999; Justi *et al.*, 2000). The champion is an Australian fruit named the Kakadu plum (*Terminalia ferdinandiana*), well known by Australian aboriginal tribes for its medicinal properties and its ascorbate content may reach up to 5 g 100 g^–1^ FW, that is 100 times that in oranges (Konczak *et al.*, 2010). Ascorbate can also vary significantly within a species, as for example in tomato (Galiana-Balaguer *et al.*, 2006) where the introduction of a wild allele by breeding resulted in increased ascorbate content in fruit (Stevens *et al.*, 2008). Also, the domestication of various fruit species has resulted in decreased ascorbate content, suggesting the occurrence of a trade-off between fruit yield and quality (Gest *et al.*, 2013). In addition to the large differences found between and within species, ascorbate concentrations vary greatly depending on the organ, tissue, cell type (Gest *et al.*, 2013), or subcellular compartment (Noctor and Foyer, 2016), but also during development (Li *et al.*, 2010; Huang *et al.*, 2014). Moreover, some studies have shown that ascorbate content changes in response to abiotic or biotic stress (Hasanuzzaman *et al.*, 2019; Grosskinsky *et al.*, 2012). Hence, it has been suggested that in response to pathogen attack, a decrease in ascorbate could lead to an oxidative burst which might prevent bacterial infection (Pavet *et al.*, 2005; Simon *et al.*, 2013). For example, in some Arabidopsis ascorbate-deficient *vtc* mutants, the reduction in the ascorbate level leads to enhanced resistance to virulent biotrophic pathogens (Mukherjee *et al.*, 2010).

It has been proposed that enhancing ascorbate production in cultivated plants could make them more resistant to stress, the frequency of which will increase due to climate change, but could also increase their nutritional value (Macknight *et al.*, 2017). However, as seen above, ascorbate levels are adjusted to a wide range of conditions, whether developmental or environmental. It is therefore not surprising that ascorbate metabolism is complex, comprising the three processes of biosynthesis, recycling, and degradation. In 1976, while the ascorbate biosynthesis pathway was still unknown in plants, the ascorbate recycling pathway, also called the Foyer–Halliwell–Asada pathway or ascorbate–glutathione cycle, was discovered (Foyer and Halliwell, 1976). Numerous studies have shown the importance of this pathway in plants growing in optimal conditions or subjected to various stresses (Foyer and Noctor, 2011). The increase in activities involved in recycling made it possible to increase the redox capacity (Badawi *et al.*, 2004; Stevens *et al.*, 2008). However, the ascorbate supply of the cycle must be continually adjusted because the products of ascorbate oxidation, monodehydroascorbate and dehydroascorbate, are unstable (Bulley and Laing, 2016), but also to adjust the total ascorbate pool according to needs. Additionally, ascorbate degradation products can play important roles (Debolt *et al.*, 2007), such as for example tartaric acid in grapes. The modalities of ascorbate biosynthesis therefore seem just as important as those of its recycling. The ascorbate biosynthetic pathway of plants, called the Smirnoff–Wheeler (SW) pathway or the l-galactose pathway, was discovered in the late 1990s (Wheeler *et al.*, 1998). In addition to the l-galactose pathway, three biosynthetic pathways have been proposed, the d-galacturonic acid pathway (Agius *et al.*, 2003), the l-gulose pathway (Wolucka and Van Montagu, 2003), and the *myo*-inositol pathway (Lorence *et al.*, 2004). Subsequently, a consensus emerged that the main pathway for ascorbate production is the l-galactose pathway, the other three probably being induced in specific developmental conditions or in specialized tissues or organs (Smirnoff, 2000; Linster *et al.*, 2007; Bulley and Laing, 2016). Since its elucidation, the regulation of ascorbate biosynthesis has been intensively investigated (Bulley and Laing, 2016) and a large number of articles describe the multiple regulatory mechanisms acting at the transcriptional and post-transcriptional levels on the l-galactose pathway. In this review, we will focus on GDP-l-galactose phosphorylase (GGP), its function in the pathway, its regulation, and finally its reported physiological roles regarding environmental and developmental challenges.

## GDP-l-galactose phosphorylase: the master of the ascorbate game

**Fig. 1.**
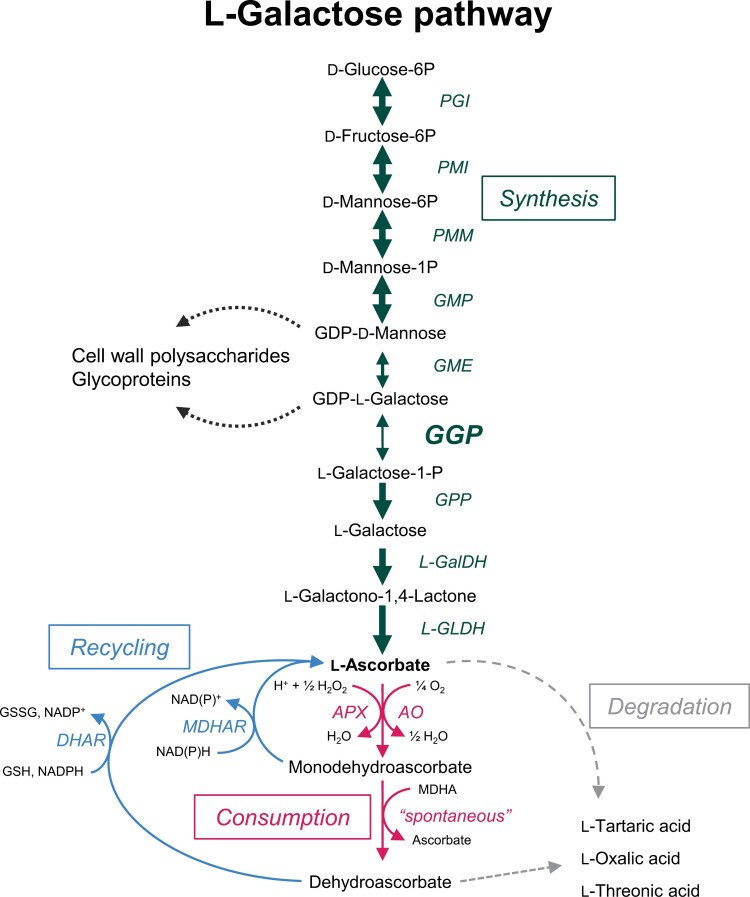
Ascorbate metabolism in plant cells. In the Smirnoff–Wheeler pathway (synthesis), the thickness of the arrows arbitrarily illustrates the capacities of the enzymes according to Fenech *et al.* (2021). AO, ascorbate oxidase; APX, ascorbate peroxidase; DHAR, dehydroascorbate reductase; GGP, GDP-l-galactose phosphorylase; GME, GDP-d-mannose-3',5'-epimerase; GMP, GDP-d-mannose pyrophosphorylase; GPP, l-galactose-1-P phosphatase; l-GalDH, l-galactose dehydrogenase; l-GLDH, l-galactono-1;4-lactone dehydrogenase; GSH, reduced glutathione; GSSG, disulfide glutathione; MDHA, monodehydroascorbate; MDHAR, monodehydroascorbate reductase; PGI, phosphoglucose isomerase; PMI, phosphomannose isomerase; PMM, phosphomannose mutase.

The SW pathway starts with d-glucose followed by several hexose phosphate intermediates. Further downstream, at the level of GDP-d-mannose and GDP-l-galactose, the pathway is connected to the biogenesis of glycoproteins and non-cellulosic compounds of the cell wall (Fig. 1). Surprisingly, GGP was the last enzyme in the pathway to be characterized. This discovery was made independently by three teams: in kiwifruit (Laing *et al*., 2007), which is known for its high vitamin C content (Ferguson and MacRae, 1992), and in ascorbate-deficient *Arabidopsis thaliana* mutants (Linster *et al.*, 2007; Dowdle *et al.*, 2007) isolated in an ozone screen (Conklin *et al.*, 2000). GGP catalyses the reversible conversion of GDP-l-galactose to l-galactose-1-phosphate in the presence of inorganic phosphate, the first specific reaction of ascorbate biosynthesis. However, although being specific for GDP-l-galactose, this enzyme is also capable of using other substrates with a lower affinity such as GDP-d-glucose, which greatly facilitates its study since GDP-l-galactose is not commercially available (Dowdle *et al.*, 2007). The genes encoding the enzymes of the SW pathway have been intensively studied, in particular by overexpression approaches. It emerged that GGP plays a central role in the synthesis of ascorbate (Bulley and Laing, 2016). Many plant species contain two genes encoding GGP, namely *VTC2* (AT4G26850) and *VTC5* (AT5G55120) in Arabidopsis, and their orthologues in kiwifruit, GGP3 (DTZ79_29g10040) and GGP1 (DTZ79_17g07300), and in tomato GGP1 (Solyc06g073320) and GGP2 (Solyc02g091510), respectively. The two GGPs of the three species share between 60% and 70% identity, in both the nucleic acid and the amino acid sequence. In Arabidopsis, *VTC2* is 100- to 1000-fold more highly expressed than *VTC5*, which is also the case for the kiwifruit and tomato homologues. Furthermore, among all the genes involved in the SW pathway, *VTC2* (or *GGP1*) has the highest expression (Dowdle *et al.*, 2007; Laing *et al.*, 2007; Massot *et al.*, 2012). Intriguingly, whereas transcriptomic data obtained in Arabidopsis rosettes and in developing tomato fruit indicate that *VTC2* (or *GGP1*) is among the 2–3% of the most highly expressed genes (Bläsing *et al.*, 2005; Belouah *et al.*, 2019), the GGP protein was not detected by proteomics in the same tissues (Uhrig *et al.*, 2020; Szymanski *et al.*, 2017; Belouah *et al.*, 2019). Localization experiments using fused proteins with green fluorescent protein (GFP) tagging in *Nicotiana benthamiana* confirmed that the abundance of GGP is very low, unlike others enzymes of the SW pathway (Fenech *et al.*, 2021). Logically, among the numerous attempts to increase ascorbate in plants by manipulating enzymes of the SW pathway (Laing *et al.*, 2007; Bulley *et al.*, 2009; Yoshimura *et al.*, 2014; Fenech *et al.*, 2021), only the overexpression of GGP (VTC2) led to a significant increase (Laing *et al.*, 2007; Bulley *et al.*, 2009, 2012; Wang *et al.*, 2014; Broad *et al.*, 2020; Chaturvedi *et al.*, 2023; Liu *et al.*, 2023). Co-overexpresion in Arabidopsis of the kiwifruit GGP and GDP-d-mannose epimerase (GME), the step which precedes GGP in the SW pathway, resulted to a 7-fold increase in ascorbate, compared with a 4-fold increase with GGP alone (Bulley *et al*., 2009). However, as quantified by Fenech *et al.* (2021), such a remarkable increase was less when using transient co-expression of these two genes in *N. benthamiana* and *A. thaliana* mutants. During tomato fruit development, levels of GGP and GME transcripts and ascorbate were found to increase concomitantly (Bulley *et al.*, 2009), suggesting that the expression of these two enzymes is co-regulated. Thus, the same regulatory factors were found to affect both *GME* and *GGP* expression (Zhang *et al.*, 2009; Zhang *et al.*, 2012; Hu *et al.*, 2016). Lastly, the use of a kinetic model describing the SW pathway and taking into account the lower capacity of GGP compared with other enzymes in the pathway as well as a negative feedback exerted by ascorbate on GGP confirmed that it is this enzyme that has by far the most control (Fenech *et al.*, 2021).

As the first step specific to ascorbate biosynthesis, GGP could also regulate the carbon fluxes dedicated on the one hand to the production of ascorbate and on the other hand to the supply of cell wall precursors (Fig. 1). Indeed, GDP-l-galactose, substrate of GGP, GDP-l-mannose, substrate of GME, and GDP-l-fucose that results from a dehydration and an epimerization of GDP-d-mannose (Bonin *et al.*, 1997), all enter into the composition of non-cellulosic constituents of the cell wall such as mannan-type hemicelluloses and the pectin rhamnogalacturonan II (Bonin *et al.*, 1997; Voxeur *et al.*, 2011). Light plays a predominant role in controlling the expression of the *VTC2* gene as well as to some extent that of the GME gene, and more particularly regarding the expression of *VTC2* that is controlled by the circadian clock (Dowdle *et al.*, 2007). Interestingly, in leaves of Arabidopsis and tomato, the transcription of *VTC2* (*GGP1*) is maximal at the end of the night, which suggests that the regulation of ascorbate biosynthesis anticipates the start of photosynthesis related to the increase of light intensity (Dowdle *et al.*, 2007; Bournonville *et al.*, 2023). Finally, Müller-Moulé (2008) found that the VTC2 protein has both a cytosolic and a nuclear subcellular localization in *A. thaliana*, a result that has since been confirmed in *N. benthamiana* (Fenech *et al.*, 2021). A first hypothesis is that in addition to its biosynthetic function, GGP could play a role in regulating the transcription of SW pathway genes (Müller-Moulé, 2008). A second hypothesis, based on the evidence that enzymes of the SW pathway co-immunoprecipitate, is the existence of an enzymatic complex enabling the channelling of the SW pathway intermediates (Fenech *et al.*, 2021), namely a metabolon (Srere, 1985).

The fact that GGP is the enzyme with by far the most control in the ascorbate synthesis pathway implies that its regulation is of great importance for the control of ascorbate concentration. We will therefore successively consider the modalities of transcription of the genes encoding this enzyme, then its translation, before addressing its post-translational regulation by light.

## Transcriptional regulation of the highly expressed GDP-l-galactose phosphorylase

Several transcription factors involved in the transcriptional regulation of *GGP* have been identified and characterized in various plant species. Thus, in kiwifruit, AcePosF21, a basic leucine zipper domain (bZIP) transcription factor, was found to increase the expression of AceGGP3 (the less abundant isoform of GGP), in response to cold stress by interacting with the R2R3-MYB transcription factor AceMYB102 that directly binds to the promoter of *AceGGP3* (Liu *et al*., 2023). It was also found that abscisic acid (ABA) inhibits the AceGBF3–AceMYBS1–AceGGP3-mediated regulation (Liu *et al.*, 2022). In tomato, SlHZ24, which belongs to the family of HD-Zip I type proteins and whose expression is induced by light, binds to the promoters of *SlGGP1* but also of *SlGMP3* (for GDP-d-mannose phosphorylase) and *SlGME2*, suggesting a multi-targeted regulation of ascorbate biosynthesis (Hu *et al.*, 2016). Still in tomato, a yeast one-hybrid screening with the SlGME1 promoter identified SlNFYA10, a CCAAT-box transcription factor that belongs to the Nuclear Factor Y (NFY) family, as a negative regulator of the expression of *SlGGP1* and *SlGME1* (Chen *et al.*, 2020). Another example of multi-targeting of the SW pathway was found in maize (*Zea mays*) where ZmbHLH55, a member of the basic helix–loop–helix (bHLH) family, promotes the expression of *ZmPGI2*, *ZmGME1*, and *ZmGLDH*, but represses *ZmGMP1* and *ZmGGP* (Yu *et al.*, 2021; Zhang *et al.*, 2012). A further example of multi-targeting was found in Arabidopsis where the overexpression of the ethylene response factor AtERF98, which was shown to bind to the promoter of *VTC1* (*GMP1*), resulted in increased expression of *VTC1*, *VTC2*, *GalDH*, and *GLDH* (Zhang *et al.*, 2012). Also in Arabidopsis, ethylene promotes *VTC2* transcription and ascorbate biosynthesis via ETHYLENE-INSENSITIVE3 (EIN3) and ABA INSENSITIVE4 (ABI4). ABI4 binds to the *VTC2* promoter to suppress *VTC2* transcription. *ABI4* is transcriptionally repressed by EIN3 in the presence of ethylene (Yu *et al.*, 2019). Salinity stress induced *ABI4* and reduced *VTC2* expression (Kakan *et al.*, 2021). In addition, the AMR1 protein (Ascorbic acid Mannose pathway Regulator 1), which is not considered to be a transcription factor and belongs to the F-box protein family linked to the SCF complex of the 26S proteasome (Moon *et al.*, 2004), is the first to have been identified in Arabidopsis as a repressor of the expression of almost all genes of the SW pathway, including *GGP* (*VTC2*) (Zhang *et al.*, 2009). This study showed that in the leaf, the expression of *AMR1* varies depending on the stage of development and light intensity (Zhang *et al.*, 2009).

Interestingly, the study of the transcriptome of acerola fruit also revealed the coordination of the expression of genes involved in ascorbate metabolism, and for some of them (e.g. GMP, GME, GGP, GalDH, and GLDH) a very high expression (dos Santos *et al.*, 2019), as is the case in Arabidopsis (Bläsing *et al.*, 2005). Indeed, transcriptomic data obtained during a day–night cycle in Arabidopsis (Bläsing *et al.*, 2005) or throughout fruit development in tomato (Belouah *et al.*, 2019) indicate that the concentrations of transcripts encoding SW pathway enzymes are high or even very high (Bournonville *et al.*, 2023). The fact that the enzymes of the pathway other than GGP are detectable (Fenech *et al.*, 2021) therefore implies the existence of drastic post-transcriptional regulation exerted on GGP.

## Translational regulation of GDP-l-galactose phosphorylase: a matter of feedback

Upstream ORFs (uORFs) are regions located upstream of the main coding region of proteins that can be translated. Initially discovered in viruses (Fütterer *et al.*, 1993), uORFs are in fact present for a large proportion of genes (more than a third in Arabidopsis) in all eukaryotes (von Arnim *et al.*, 2014). Initiation of translation from a uORF can repress translation from a downstream ORF by blocking ribosome movement. Furthermore, if the uORF stop codon is located upstream of the exon junction complex, it can be recognized as a premature termination codon and thus activate mRNA degradation. A predominant idea is that uORFs are rather associated with genes involved in development and responses to stress, whereas so-called housekeeping genes lack them (von Armin *et al.*, 2014). Consistent with the role of ascorbate in stress responses, a highly conserved uORF has been found for GGP isoforms in a wide range of plant species (Laing *et al.*, 2015). It has been shown in Arabidopsis (Laing *et al.*, 2015) as well as in tomato (Li *et al.*, 2018; Deslous *et al.*, 2021) that mutations in this region lead to ascorbate-enriched phenotypes. In other words, the increase in GGP activity resulting from the de-repression of its translation would lead to more ascorbate, in agreement with the strongly controlling role of this enzyme proposed by Fenech *et al.* (2021). Furthermore, the repression of GGP translation by its uORF only occurs at high ascorbate concentrations (Laing *et al.*, 2015). It is therefore a feedback mechanism, the modalities of which are not yet completely elucidated. The peptide encoded by the GGP uORF would be involved because a change in its amino acid sequence eliminates the feedback. To date, very few of such peptide-conserved uORFs have been discovered, but all are believed to be involved in responses to stress (Causier *et al.*, 2022). We have seen above that the GGP protein levels are very low while the mRNAs that encode it are among the most abundant. This discrepancy could be, at least in part, explained by this feedback mechanism.

## Post-translational regulation of GDP-l-galactose phosphorylase is a LOV story

The stimulation of ascorbate synthesis by light has been known for some time. Arabidopsis leaves contained three times more ascorbate when grown under 250 µmol^–1^ m^–2^ s^–1^ light intensity than when grown under 50 µmol^–1^ m^–2^ s^–1^ (Bartoli *et al.*, 2006). Similar results were obtained with tomato fruit (Zhang *et al.*, 2021), including at the breaker stage, suggesting that photosynthesis is not necessarily involved. Moreover, still in tomato, ascorbate was significantly reduced in fruit growing in the dark while sugars (its precursors) were not affected (Gautier *et al.*, 2009). Interestingly, various light treatments tested on plants or fruits have shown that it is blue light that most promotes ascorbate accumulation (Li *et al.*, 2022; Ganganelli *et al.*, 2023). Various explanations have been proposed for the effect of blue light on ascorbate synthesis, but recent work suggests that it is mainly due to post-translational regulation involving a photoreceptor called PAS/LOV (Aarabi *et al.*, 2023; Bournonville *et al.*, 2023).

Plants use blue light to perceive, via photoreceptors including phototropins, members of the ZEITLUPE family, and cryptochromes (Christie *et al.*, 2015), the quantity of light energy available in order to optimize their development, but also to protect themselves from excessive light, in particular thanks to the different levels of photosensitivity of these photoreceptors (Gyula *et al.*, 2003; Christie, 2007). Thus, blue light represses the growth of the hypocotyl and internodes, which makes shoots more compact, promotes leaf thickness, and induces flowering as well as the production of secondary compounds such as carotenoids and flavonoids (Huché-Thélier *et al.*, 2016). The idea that the perception of blue light induces protective mechanisms is reinforced by the fact that the flavins that generate ROS when elicited by blue light are precisely the cofactors of these flavoproteins (Losi and Gärtner, 2012).

The light oxygen voltage-sensitive (LOV) domain, which is part of the Per-aryl hydrocarbon receptor nuclear translocator Sim (PAS) superfamily, is a ubiquitous photoreceptor (Glantza *et al.*, 2016). Blue light initiates the formation of a covalent bond between a flavin (most often FMN) and a cysteine residue, which causes a conformational change in the protein that will initiate signal transduction. The modular nature of LOV domains has allowed the emergence of a large number of photoreceptors (Glantza *et al.*, 2016). Among them, phototropin, which was discovered in Arabidopsis in 1997 (Huala *et al.* 1997), has been intensively studied. It has two LOV domains (LOV1 and LOV2) in the N-terminal region and a serine-threonine kinase domain in the C-terminal region. Once activated, it initiates various responses such as phototropism, stomatal opening, chloroplast movements, and leaf expansion and movements (Christie, 2007). The PAS/LOV protein, mentioned for the first time in plants in 2003 (Crosson *et al.*, 2003), would come from a protein that also had two LOV domains, but whose LOV1 domain would no longer be functional following the replacement of one cysteine by a glycine in a conserved site (Kasahara *et al.*, 2010). This is why this protein is also called LOV/LOV. By using a yeast two-hybrid approach, Ogura *et al.* (2008) showed that PAS/LOV interacts with two members of the BEL1-like homeodomain protein family (BLH10A and BLH10B), which are transcription factors involved in many aspects of development (Ezura *et al.*, 2022), and with GGP. The fact that such a receptor interacts directly with an enzyme of a synthetic pathway is unusual, as most interact with transcription factors. It is only very recently that this interaction was shown *in vivo* and *in vitro* to be responsible for the inhibition of GGP, which was found to be inactivated by blue light (Bournonville *et al.*, 2023).

One may wonder why the elucidation of this mechanism took so long once the interaction between PAS/LOV and GGP was known. Perhaps the reverse genetics approach of studying PAS/LOV mutants would have been unsuccessful due to the low light intensity under which Arabidopsis is usually grown. In fact, this elucidation was achieved thanks to forward genetics. Thus, a tomato mutant enriched in ascorbate due to a truncated PAS/LOV was found by screening a population of ethyl methanesulfonate (EMS) mutants grown in a greenhouse (Bournonville *et al.*, 2023). Around the same time, another team discovered the role of PAS/LOV with another forward genetics approach, genome-wide association study (GWAS), using an Arabidopsis diversity panel (Aarabi *et al.*, 2023). Interestingly, these authors found quantitative trait loci (QTL; notably located in the PAS/LOV promoter) for the increase in the leaf of ascorbate level caused by a strong increase in light intensity.

*In vitro* experiments with recombinant GGP and PAS/LOV showed that PAS/LOV is a non-competitive inhibitor of GGP (Bournonville *et al.*, 2023). Blue light counteracted this inhibition, but only when applied before mixing the two proteins, suggesting that the inhibition is irreversible. *In vivo*, it would therefore be the newly formed PAS/LOV that is deactivated by blue light. As for other proteins with LOV domains, light induces a rapid change in the conformation of PAS/LOV (Kasahara *et al.*, 2010), significantly faster than its return to the dark form. Measurements of GGP inhibition by PAS/LOV corroborated these results, showing in particular that blue light deactivation of PAS/LOV was stable for at least 6 h. Another striking result was that the time required to deactivate PAS/LOV decreased as the intensity of blue light increased, until reaching a plateau at ~200 µmol m^–2^ s^–1^, an intensity that corresponds to the amount of blue light present in sunlight (Bournonville *et al.*, 2023).

There are still open questions about the interaction between PAS/LOV and GGP. Thus, the two interacting proteins were localized by bimolecular fluorescence complementation (BiFC) in the cytosol as would be expected, but also in the nucleus and the peroxisome (Aarabi *et al.*, 2023; Bournonville *et al.*, 2023). Noting up-regulation of *VTC2* in *plp* Arabidopsis mutants, Aarabi *et al.* (2023) raised the possibility of the existence of a negative feedback by which this complex would repress *GGP* transcription. However, the opposite was observed, with a decrease in the expression of *GGP1* in a *plp* tomato mutant (Bournonville *et al.*, 2023). Another possibility would be the targeting via ubiquitination of the complex towards the 26S proteasome located in the nucleus (Bournonville *et al.*, 2023), similar to what has been reported with ZEITLUPE, which is another LOV domain photoreceptor targeting transcription factors (Yasuhara *et al.*, 2004; Kiba *et al.*, 2007; Seo *et al.*, 2023). A further intriguing point is that many copies of the PAS/LOV protein were necessary to inhibit GGP activity *in vitro*, but it is not known whether this was because of a conformational problem due to heterologous expression. It would therefore be interesting to crystallize the PLP/GGP complex in order to better understand its stoichiometry. Indeed, the latter can be expected to play a major role in controlling ascorbate synthesis. Moreover, it will undoubtedly be important to characterize the turnover of these proteins whose genes are mainly expressed during the night in the leaves. Also, it is striking that PAS/LOV appeared as one of the top sugar-repressed genes in Arabidopsis (Bournonville *et al.*, 2023).

## Modulating GDP-l-galactose phosphorylase impacts plant physiological responses

Given the importance of ascorbate as an integral player in plant redox metabolism, it is clear that modulation of GGP with subsequent impacts on ascorbate levels results in important physiological phenotypes, the most relevant of which are described in this section.

As already extensively reviewed, environmental fluctuations influence ascorbate levels, which in turn exhibit evident connections with GGP (Table 1) (Decros *et al.*, 2019; Broad *et al.*, 2020). Several studies in various species report an effect of light on ascorbate content: levels rise in the light and plummet in the dark (Castro *et al.*, 2023). Interestingly, tomato genes involved in the l-galactose pathway respond to light owing to light-responsive promoter elements, including six motifs in the case of GGP (Ioannidi *et al.*, 2009). Indeed, the most prevalent motifs found in the promoter sequences were those responsive to light, alongside elements responsive to stress signals such as ABA, gibberellin, heat shock, wounding, fungal elicitors, and endosperm. Further research in Arabidopsis supports the light dependence of ascorbate synthesis, as exemplified by *vtc3* mutant plants that exhibited a deficiency in their capacity to increase ascorbate in response to light and heat (Conklin *et al.*, 2013). The recent revelation of how the PAS/LOV blue light photoreceptor (described in the previous section) directly interacts with GGP and inhibits ascorbate production has enhanced our comprehension of the connection between ascorbate and light (Bournonville *et al.*, 2023). Y.Z. Zhang *et al.* (2023) found that overexpression of GGP combined with a reduction in *PAS/LOV* expression made soybean plants more stress-resistant. The same team studied the *PLP1* promoter, showing that it was up-regulated under conditions of darkness, blue light, gibberellin A3, and ABA. The strongest stimulation was observed at the seed and seedling stages, while promoter activity decreased as the plant neared maturity. These results confirm the regulation of the *PLP1* promoter in developing seedlings and seeds (Luo *et al.*, 2013). In terms of plant physiology, the promoting effect of blue light on ascorbate synthesis would enable the plant to cope with the increase in ROS production when light intensity increases. In addition, higher ROS content tends to increase the proportion of oxidized forms of ascorbate (Decros *et al.*, 2019), which are less stable (Bulley and Laing, 2016). The fact that PAS/LOV expression is repressed by sugars (Bournonville *et al.*, 2023), themselves the products of photosynthesis, further indicates the existence of a trade-off between growth and defence. Indeed, on the one hand, the synthesis of ascorbate occurs to the detriment of the use of sugars for growth, and, on the other hand, the accumulation of sugars in leaves can be seen as reflecting an excess of energy. However, this remains to be clearly established. While ascorbate may have concentrations comparable with those of Suc/Glc, numerous publications suggest that its turnover is slower. Sugars, on the other hand, are rapidly replenished, resulting in distinct fluxes.

**Table 1.** Physiological effects of altered GGP expression in transgenic and mutant plants

Study	Species	Genotype	Impact on ascorbate levels	Phenotype	Reference
Growth and development	Arabidopsis	*vtc2-1-vtc5*	90% of wild type in the double mutant	Growth arrest after germination, cotyledon bleaching	Dowdle *et al*. (2007); Lim *et al.* (2016)
	Arabidopsis	*vtc2-1*, *vtc2-2*, *vtc2-4*, and *vtc2-5*	10–20% of wild type	Smaller size	Dowdle *et al*. (2007); Lim *et al*. (2016); Plumb *et al*. (2018)
	Arabidopsis	*vtc2-3*	50% of wild type	Similar to wild type	Dowdle *et al*. (2007)
	Arabidopsis	*vtc5*	90% of wild type	Normal phenotype	Dowdle *et al*. (2007)
	Tomato	*slggp1*	Lower than wild type	High light sensitive	Baldet *et al.* (2013)
	Banana	*GGP OE*	150–200% of wild type	Improved plant growth	Chaturvedi *et al*. (2023)
	Tomato	*uORF-GGP1*	500% of wild type	Impaired floral architecture (pollen sterility), seedless fruit	Deslous *et al.* (2021)
	Arabidopsis	*VTC2 OE* (pollen specific)	No increase in ascorbate or lower redox potential in mature pollen grains	Reduced fertility	Weigand *et al.* (2023)
Light responses	Multiple	WT	Increase	Increased GGP mRNA	Castro *et al*. (2023)
Light responses	Tomato	*plp* CRISPR/Cas9	200–300% of wild type	No phenotype reported	Bournonville *et al.* (2023)
Light and heat stress	Arabidopsis	*vtc3*	Decrease	No significant difference in ascorbate redox status, defective in heat- and light-induced ascorbate elevation	Conklin *et al*. (2013)
Ozone sensitivity	Arabidopsis	*vtc2-1*, *vtc2-2*, and *vtc2-3*	Decrease	Increased ozone sensitivity	Conklin *et al*. (2000)
Cold stress	Kiwifruit	Wild type, validation using VIGS and CRISPR/Cas9	Increase	Cold-responsive *AceGGP3* up-regulation to mitigate oxidative damage caused by cold stress	Liu *et al.* (2023)
Cold and bacterial stress	Tomato	As *GGP-LIKE*	Decrease	Growth inhibition, higher ROS, ion leakage, and malondialdehyde under chilling stress, with lower bacterial infection	Yang *et al.* (2017)
Fungal infection	*Vitis sp*	*GGP-LIKE OE*	Increase	Defence elicitation including defence molecules	Hou *et al*. (2013)
Viral infection	Wheat	*VTC2 OE/VTC2-RNAi*	Decrease in *VTC2-RNAi*	ROS burst in *VTC2-RNAi* leaves	T.Y. Zhang *et al.* (2023)

Considering cold stress, *AcePosF21*, a bZIP transcription factor in kiwifruit (*Actinidia eriantha*), was found to mitigate oxidative damage caused by stress via up-regulation of *AceGGP3* expression and thus ascorbate biosynthesis (Liu *et al.*, 2023). Conversely, when *AcePosF21* was silenced through virus-induced gene silencing (VIGS) or edited using CRISPR/Cas9 [clustered regularly interspaced palindromic repeats (CRISPR)/CRISPR-associated protein 9], ascorbate levels decreased, resulting in elevated ROS production in kiwifruit under cold stress. Still in the context of temperature tolerance, a study in tomato suggests that the *SlGGP-LIKE* gene, which encodes the less abundant isoform of GGP, plays a role in plant defence against chilling stress and pathogenic infection (Yang *et al.*, 2017). Antisense transgenic lines, in which this gene was targeted, showed lower ascorbate levels compared with wild-type plants and experienced significant growth inhibition, and higher levels of ROS, ion leakage, and malondialdehyde under chilling stress. Other physiological phenotypes were impacted, such as significant declines in photosynthesis, PSII efficiency (*F*_v_/*F*_m_), oxidizable P700, and D1 protein content, together with further severe changes in the xanthophyll cycle. Moreover, even under chilling stress, the antisense plants infected with the *Pseudomonas syringae* pv. *tomato* (*Pst*) DC3000 strain showed reduced bacterial growth and cell damage compared with wild-type plants, demonstrating improved resistance to pathogenic infection (Yang *et al.*, 2017). Furthermore, expression of the *GGP-LIKE* gene in a Chinese wild *Vitis* species elicits reactions to pathogenic *Erysiphe necator*, and signals the activation of defence molecules, including salicylic acid, methyl jasmonate, and ethephon (Hou *et al.*, 2013). Notably, this research also revealed a strong correlation between *VTC* transcripts and the level of disease resistance in three distinct genotypes. In a different pathosystem, a recent study indicated that *VTC2* up-regulation in wheat substantially boosted viral accumulation of wheat yellow mosaic virus (WYMV), while *VTC2* down-regulation hindered viral infection (T.Y. Zhang *et al.*, 2023). Furthermore, in *VTC2*-*RNAi* plants, lower ascorbate production occurred due to reduced *VTC2* expression and activity. This reduction in ascorbate levels led to increased ROS in the leaves. In this context, GGP probably augments ascorbate, so the effect on viral infection is due either to ascorbate itself or to increased ROS removal. As with many plant–microbe interactions, other signalling processes could be involved and the different correlations reported between disease and GGP expression/ascorbate levels could be due to the different mechanisms activated by the different pathogens tested. Further analyses are therefore essential to clarify the nature and role of signalling processes.

Besides stress responses, the physiological roles involving GGP also concern plant development, particularly from very early developmental stages. It has been shown in tomato that increased ascorbate synthesis via the loss of GGP retroinhibition by uORF led to parthenocarpic-like fruit, which was attributed to an impairment of pollen fertility with deleterious pollen germination rates (Deslous *et al.*, 2021). This phenotype was further accompanied by an affected transcriptome in pollen tissues, notably in relation to the down-regulation of pollen genes *SlMS10*, *SlINO*, *SlSES*, and *SlEMS/EXS*. In the same investigation, the study verified the expression of parthenocarpy genes and suggested that the seedless trait observed in the fruits of the ascorbate-enriched mutants was not due to parthenocarpy. Instead, it was more likely to be linked to a broader issue of male sterility, potentially arising from disruptions in multiple processes occurring during pollen development, possibly redox mechanisms (Deslous *et al.*, 2021). Congruently, certain stages of development require increased ROS, in particular the destruction of the tapetum in anthers, a step that is essential for the production of fully developed pollen (Yi *et al.*, 2016). Similarly, a recent investigation in Arabidopsis revealed that the specific overexpression of *GGP* (*VTC2*) within pollen tissues resulted in decreased fertility, accompanied by changes in tip-focused Ca^2+^ dynamics (Weigand *et al.*, 2023). Strikingly, pollen overexpressing *VTC2* did not exhibit the anticipated increases in ascorbate levels or the reduction in ROS. On the other hand, the expression of two enzymes of the SW pathway, GMP and GME, which are also involved in wall synthesis, was repressed. As in tomato (Deslous *et al.*, 2021), this study in Arabidopsis confirms that the reduction in pollen fertility links to an alteration in biosynthetic pathways related to cell walls. Intriguingly, in the tomato knockout *Slggp1* mutant, fertility was not impacted even though the ascorbate content was significantly decreased (Baldet *et al.*, 2013). Furthermore, analysis of Arabidopsis mutants affected in VTC2 and VTC5 indicates a range of developmental phenotypes linked to reduced ascorbate levels (Table 1) (Dowdle *et al.*, 2007; Lim *et al.*, 2016). While *vtc2-1×vtc5* double mutation resulted in a growth arrest after germination, followed by the bleaching of cotyledons after 2 weeks, *vtc2*-1 and *vtc2-2* single mutants had a smaller size, and *vtc2-3* and *vtc5* displayed no size phenotype compared with wild-type plants. Conversely, a study aiming at the biofortification of plants by ascorbate showed that overexpressing banana GGP in Arabidopsis led to improved plant growth (Chaturvedi *et al.*, 2023). A recent review also highlights that growth processes in various species (tobacco, *Malpighia glabra* ‘acerola’, *Brassica campestris*, *Citrus unshiu*, *C. sinensis*, and *Myrciaria dubia* ‘camu-camu’) are underpinned by transcriptional regulation of several ascorbate synthetic genes, including GGP (Castro *et al.*, 2023). Hence, although a clear link exists between ascorbate levels and *GGP* expression, the diverse range of growth phenotypes implies that additional pleiotropic effects, potentially stemming from redox and signalling changes, play a role in the observed developmental disruptions. Studying environmental cues in the context of ascorbate synthesis corroborates this idea.

Overall, GGP certainly fuels ascorbate which is at the heart of at least three trade-offs, namely between (i) growth and defence against excess light; (ii) defence against abiotic and biotic stress; and (iii) defence and development. However, the evidence supporting such roles for GGP remains relatively scarce and fragmentary, requiring further research to establish the precise contributions. To address this gap, metabolic models that couple ascorbate synthesis with the ascorbate–glutathione (GSH) cycle would help provide mechanistic data to advance our understanding (Fenech *et al.*, 2021; Decros *et al.*, 2023). By exploiting kinetic modelling, a sequential metabolic regulation of redox fluxes could be established in developing tomato fruit, which depended on the developmental stage and involved ascorbate synthesis, NAD(P)H levels, and ROS availability (Decros *et al.*, 2023). This study also points out that oxidized ascorbate appears important for the early stages of fruit development since the highest value was found at the beginning of fruit development before falling rapidly and increasing, to a lesser extent, during early ripening. This overoxidation of ascorbate could be the result of an intensive metabolic rate in very young fruit, which transiently alters the redox ratio. Furthermore, ascorbate content in the fruit is mainly controlled by its own synthesis rather than by import from the leaves, as is the case for photoassimilates and despite a light-dependent regulation mechanism (Gautier *et al.*, 2009; Bournonville *et al.*, 2023). These findings underscore the value of metabolic models in gaining a deeper understanding of the connection between ascorbate synthesis and redox regulations. Other innovative technologies will further enable us to better pinpoint the signals involved in these molecular interactions, especially metabolic imaging, which will provide spatial and tissue-specific distributions of ascorbate forms and their metabolic partners.

## Conclusion

**Fig. 2.**
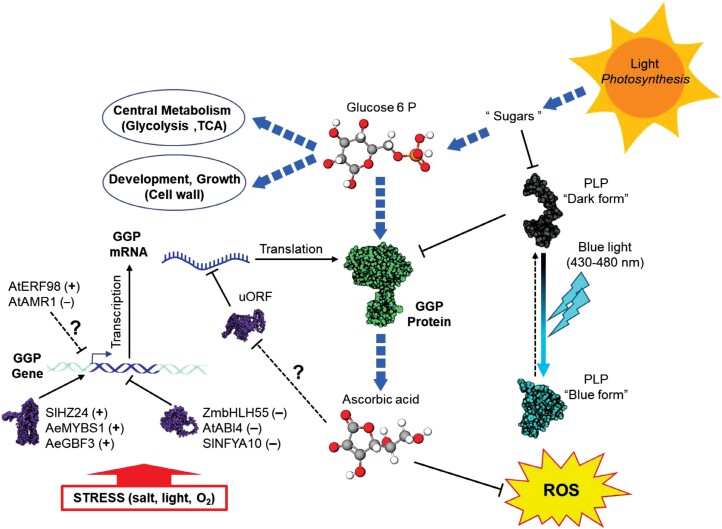
Model of regulation of the GDP-l-galactose phosphorylase (GGP). GGP is regulated at the transcriptional, post-transcriptional, and protein levels. The proteins AtAMR1 and AtER98 are a repressor and activator of GGP expression, respectively, but their mode of action is still unknown (Zhang *et al.*, 2009; Zhang *et al.*, 2012). Several transcription factors recognize and bind specific *cis*‐acting elements of the promoter to control the expression of the *GGP* gene. Among them, SlHZ24 (Hu *et al.*, 2016), AeMYBS1 (Liu *et al.*, 2022), AeGBF3 (Liu *et al.*, 2022), AtABI4 (Yu *et al.*, 2019), SlNFYA10 (Chen *et al.*, 2020), and ZmbHLH55 (Yu *et al.*, 2021) are activators (+) or repressors (–) and both are induced under various stress conditions. GGP is also repressed at the post-transcriptional level by a *cis*-acting upstream ORF (uORF) located in the 5'-untranslated region of its mRNA. It was hypothesized that under high ascorbate content, the uORF inhibits the translation of the GGP mRNA and the opposite when ascorbate is low (Laing *et al.*, 2015). At the protein level, the GGP catalytic activity is inhibited by the blue light photoreceptor PAS/LOV (PLP). In the absence of light, PLP in its ‘dark form’ inhibits GGP. Under a blue light signal, the conformation of PLP changes rapidly leading to its ‘blue form’ that is unable to interact with GGP (Bournonville *et al.*, 2023). Ascorbate itself also participates in the elimination of ROS. Sugar (glucose 6P) from photosynthesis activity, in addition to being a precursor of central metabolism and plant growth, is also a precursor of the Smirnoff–Wheeler pathway, which can repress PLP expression (Bournonville *et al.*, 2023).

**Fig. 3.**
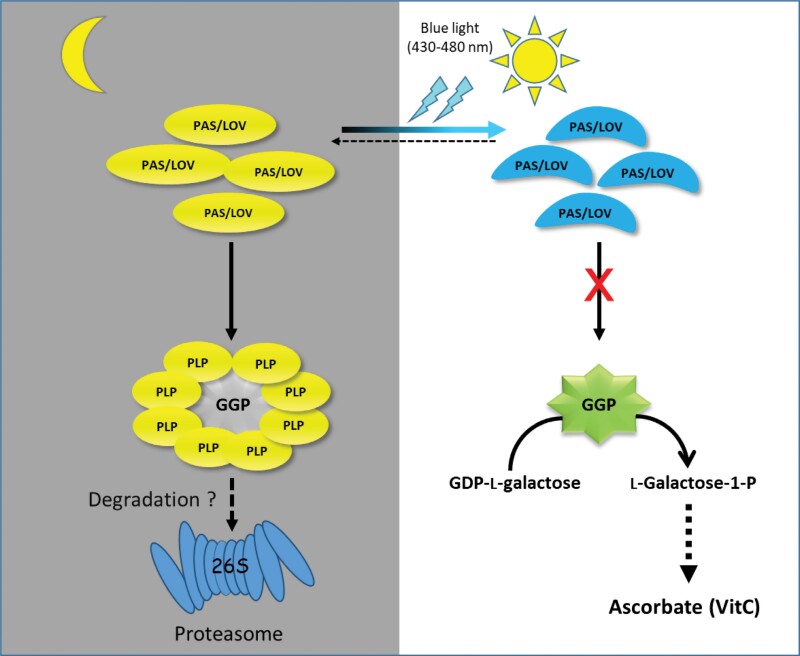
Model of regulation of ascorbate (vitamin C) biosynthesis in plants by the photoreceptor protein PAS/LOV at the level of GDP-l-galactose phosphorylase (GGP). At night or in darkness, the PAS/LOV photoreceptor adopts a conformation that allows it to bind to GGP, which leads to the inhibition of GGP activity. The formed GGP–PAS/LOV complex is stable but its fate still remains unknown. One hypothesis would be its degradation by the 26S proteasome. When the plant is exposed to the sun, the blue light component (430–480 nm) of the solar spectrum causes a reversible modification of the PAS/LOV photoreceptor. In its new conformation, PAS/LOV can no longer bind to the GGP enzyme (adapted from Bournonville *et al.*, 2023).

The pleiotropic nature of the action of ascorbate, sometimes detoxifier, sometimes cofactor, sometimes possible regulator, is reflected in the complexity of its metabolism. Its biosynthesis pathway has been intensively studied since it was elucidated 25 years ago. Although our vision is undoubtedly still fragmentary, it appears that GGP plays a major role in ascorbate metabolism. By far the least abundant enzyme in the SW pathway, it exerts powerful control over ascorbate synthesis. Strikingly, while the mRNAs that encode it are among the most abundant in the transcriptome, its post-transcriptional regulation appears drastic: its translation is subject to negative feedback, and its activity is inhibited by a photoreceptor whose action is counteracted by blue light. It will certainly be valuable to incorporate a module dedicated to GGP that considers the three levels of regulation of its expression (Fig. 2) in a model able to integrate the multiple factors influencing ascorbate metabolism. Indeed, the very strong expression of GGP can be seen as providing great flexibility in response to stress and to adequately address trade-offs. Thus, when the ascorbate concentration decreases following its oxidation, its synthesis would be stimulated more quickly than following a more complex and slower response involving both transcriptional and translational regulation steps. The mechanism involving PAS/LOV further enables a swift adjustment of ascorbate synthesis (Fig. 3). This adaptation mechanism may prove crucial during sudden changes in light conditions. The role of this double checkpoint could also be to prevent excessive ascorbate, either to avoid wasting resources or to prevent toxicity. A better understanding of how ascorbate metabolism is regulated will undoubtedly be very beneficial for the development of varieties adapted to future environmental conditions.
